# Reducing barriers to trauma inquiry in substance use disorder treatment – a cluster-randomized controlled trial

**DOI:** 10.1186/s13011-019-0211-8

**Published:** 2019-05-29

**Authors:** Annett Lotzin, Sven Buth, Susanne Sehner, Philipp Hiller, Silke Pawils, Franka Metzner, John Read, Martin Härter, Ingo Schäfer

**Affiliations:** 1 Department of Psychiatry and Psychotherapy, University Medical Center Hamburg-Eppendorf, Hamburg, Germany; 2 Center for Interdisciplinary Addiction Research, University of Hamburg, Hamburg, Germany; 3 Institute for Interdisciplinary Addiction and Drug Research, Hamburg, Germany; 4 Department of Medical Biometry and Epidemiology, University Medical Center Hamburg-Eppendorf, Hamburg, Germany; 5 Department of Medical Psychology, University Medical Center Hamburg-Eppendorf, Hamburg, Germany; 6 School of Psychology, University of East London, London, UK

**Keywords:** Addiction, Substance use disorders, Counseling, Comorbidity, Trauma-informed care, Abuse, Neglect

## Abstract

**Background:**

Despite the high rate of traumatic events in clients with substance use disorders, trauma exposure often remains undetected in a majority of treatment-seeking clients. Improving health professionals’ knowledge and skills in the inquiry of traumatic events is therefore of utmost importance for appropriately addressing trauma-related treatment needs. However, professionals in substance use disorder treatment settings frequently report barriers to the inquiry about traumatic events, e.g., the fear of offending or harming the client. Such barriers should be addressed by trainings that aim to improve the systematic inquiry of traumatic events.

**Methods:**

Using a cluster-randomized trial, we examined whether a one-day training in trauma inquiry (‘Learning How to Ask’) would reduce professionals’ perceived barriers to trauma inquiry. One hundred forty-eight professionals working in outpatient substance use disorder treatment centers were randomized to an intervention (*n* = 72) or a control group (*n* = 76). The professionals in the intervention group received a one-day training plus a refresher session 3 months later, the professionals in the control group received no training.

At baseline, and at 3-month and 6-month follow-up, professionals rated on a four-point Likert scale regarding how strongly they agreed with statements about six common barriers to trauma inquiry, namely ‘Feeling uncomfortable when asking about traumatic events’, ‘Fear of offending the client’, ‘Fear of retraumatizing the client’, ‘Fear that client may terminate treatment’, ‘Unsure whether authorities have to be informed when perpetrator is known’, and ‘No trauma-specific treatment available in my local area’.

**Results:**

The trained group experienced significant greater decreases in five of the six perceived barriers to the inquiry of traumatic events from baseline to 6-month follow-up than the control group (‘Feeling uncomfortable when asking about traumatic events’: *b* = − 0.32, 95% CI [− 0.52, − 0.12]; ‘Fear of offending the client’: *b* = − 0.33, 95% CI [− 0.56, − 0.09]); ‘Fear of retraumatizing the client’: *b* = − 0.45, 95% CI [− 0.69, − 0.22]; ‘Fear that client may terminate treatment’: *b* = − 0.28, 95% CI [− 0.49, 0.07]; *‘*No trauma-specific treatment available in my local area’: *b* = − 0.25, 95% CI [− 0.51, − 0.01]).

**Conclusions:**

Our findings provide first evidence that a one-day training in trauma inquiry is effective in reducing common barriers to trauma inquiry, which may in turn improve detection of traumatic events.

**Electronic supplementary material:**

The online version of this article (10.1186/s13011-019-0211-8) contains supplementary material, which is available to authorized users.

## Background

Health care professionals that work in substance use disorder treatment settings are frequently faced with clients that were exposed to trauma. More than half of the clients that seek substance use disorder (SUD) treatment report to have experienced traumatic events [1]. Consequently, individuals with SUD have been found to be seven times more likely to receive a diagnosis of posttraumatic stress disorder (PTSD) than individuals without SUD [2]. The highest rates of PTSD were reported for individuals with SUD that use sedatives, opioides or amphetamines [2]. The association between PTSD and SUD can be explained by the fact that substances might be helpful in the short term to reduce the arousal, distress or anxiety related to the symptoms of PTSD [3]. Compared to SUD clients without trauma exposure, SUD clients with trauma exposure have poorer SUD treatment outcomes [1, 4] and are more likely to be affected by trauma-related comorbid mental disorders that have to be considered in treatment.

Acknowledging the interdependency between trauma and SUD, a systematic assessment of trauma exposure and trauma-related symptoms is needed in SUD health care settings to appropriately address the specific trauma-related treatment needs of this vulnerable group [5, 6] according to international treatment guidelines [7–9]. The implementation of trauma-informed care in SUD settings seems essential. In particular, the implementation should include the systematic inquiry of traumatic events, an adequate response to reports of traumatic events and, if necessary, the provision of or referral to specialized services [5].

However, to date, traumatic events and related symptoms remain undetected in clients accessing mental health services in most cases [10, 11]. Only a small proportion of the clients seeking services with trauma exposure receives trauma-informed care or trauma-specific treatment in Europe [12–14].

Although most health care professionals believe that clients should be systematically screened for traumatic events because this knowledge is critical to treatment planning, they frequently fail to systematically inquire traumatic events in routine practice [15, 16]. Whether health care professionals inquiry traumatic events or not is related to professionals’ gender [11, 17–19], age [19–21], professional group [22] and cultural background [22, 23].

A broad range of psychological and structural barriers to systematic trauma inquiry exists in professionals across different health care settings. One of the most often reported perceived barriers to trauma inquiry is *feeling uncomfortable when asking about traumatic events* [18, 21, 24–26]. For example, health care professionals in primary care settings reported not to ask their clients for traumatic events, because they felt uncomfortable and powerless when doing so. Similarly, mental health care professionals in SUD treatment settings reported that one of their most important barriers to trauma inquiry was feeling uncomfortable when asking about traumatic events [27].

The *fear of offending the client* [18, 23, 26] is a frequently reported barrier to trauma inquiry. In a study among physicians [26], fear of offending the client was one of the strongest concerns reported. This fear was related to the thought that talking about trauma exposure was a sensitive topic that is culturally defined as private and that should only be explored if it is really needed. Similarly, health care professionals in primary care settings reported to fear offending the client when asking about traumatic events [26]. Since asking about traumatic events was perceived as a possible offence, the professionals also reported to *fear that the client may terminate treatment* when asking about such events.

Another common perceived barrier to trauma inquiry is *the fear of retraumatizing the client* [18, 21, 28]. In a qualitative study, mental health professionals reported that asking about traumatic events might ‘open up a can of worms and create perhaps re-traumatization for the client’ [21]. Similarly, health care professionals in primary care settings have been found to believe that the inquiry of trauma may ‘open Pandora’s box’ [26]. In a survey of psychologists and psychiatrists [19], reasons for not inquiring about traumatic events involved the fear of worsening the clients’ psychological symptoms.

*Lacking knowledge of relevant legislation,* e.g., whether authorities have to be informed when perpetrator is known, was reported by general practitioners as a barrier to trauma inquiry [22]. Lack of resources and referral constitute another structural barrier to trauma inquiry [22]. Within SUD health care settings, the *lack of availability of trauma-specific treatment* for SUD clients might hinder professionals to inquire traumatic events in their clients.

Barriers to trauma inquiry are closely linked to trauma-related knowledge and skills [21–24, 27, 29, 30]. Most health care professionals are not formally trained in the inquiry about traumatic events and in dealing with their own emotional response to reports of such events, as knowledge and skills are not part of their professional training. Consequently, health care professionals in SUD settings reported to feel uncomfortable when asking about traumatic events because they lacked knowledge about trauma-related symptoms and felt insuffienctly trained in trauma inquiry [27].

Unfortunately, very few trainings of trauma inquiry are available [31]. Read and colleagues [28, 32] developed a one-day ‘Learning How to Ask’ training program on the inquiry about traumatic events, and adequate response strategies to reports of traumatic events, that can be used in different health care settings. In this training, health care professionals learn basic rules of trauma inquiry and response, which are practiced in role plays. This training has been found to be effective in improving trauma-related knowledge and skills in a pilot study [32]. The training was recently adapted and evaluated for SUD treatment settings in a cluster-randomized controlled trial by our research group [33]. The results of this study provided evidence that the training might be effective in increasing the inquiry of traumatic events [33]. However, it has been not examined whether this training was effective in reducing common perceived barriers to trauma inquiry. Therefore, the aim of this secondary analysis of the data of this trial was to examine whether the one-day ‘Learning How to Ask’ training in trauma inquiry, combined with a booster session after 3 months, is effective in reducing professionals’ perceived barriers to trauma inquiry. We assumed that six frequently reported barriers to trauma inquiry (‘*Fear of offending the client’, ‘Fear of retraumatizing the client*’, ‘*Fear that client may terminate treatment*’, ‘*Feeling uncomfortable when asking about traumatic events*’, ‘*Unsure whether authorities have to be informed when perpetrator is known*’, ‘*No trauma-specific treatment available in my local area*’) would decrease, from baseline to 6-month follow-up, in a group of trained professionals relative to a group of untrained professionals.

## Methods

### Design

This research is part of a larger cluster-randomized trial [33] that was designed to evaluate the effectiveness of a training in the inquiry of traumatic events. The study was approved by the local ethics committee (‘Ethikkommission der Ärztekammer Hamburg’) and was conducted in accordance with the Declaration of Helsinki [34]. This study is not a clinical trial, i.e., a study that prospectively assigns human participants to health-related interventions to evaluate the effects on health outcomes [35], and has therefore not been registered in a clinical trial register.

### Participants and setting

One hundred forty-eight health care professionals of 27 German SUD outpatient treatment centers of the federal states Hamburg and Schleswig-Holstein were included in the study. To be included, SUD centers had to offer outpatient service for clients with SUD. Health care professionals from the SUD centers were included in the study if they were providing counseling or outpatient therapy for SUD clients, i.e., if they were in direct contact with clients, and if there were willing to participate.

**Fig. 1:**
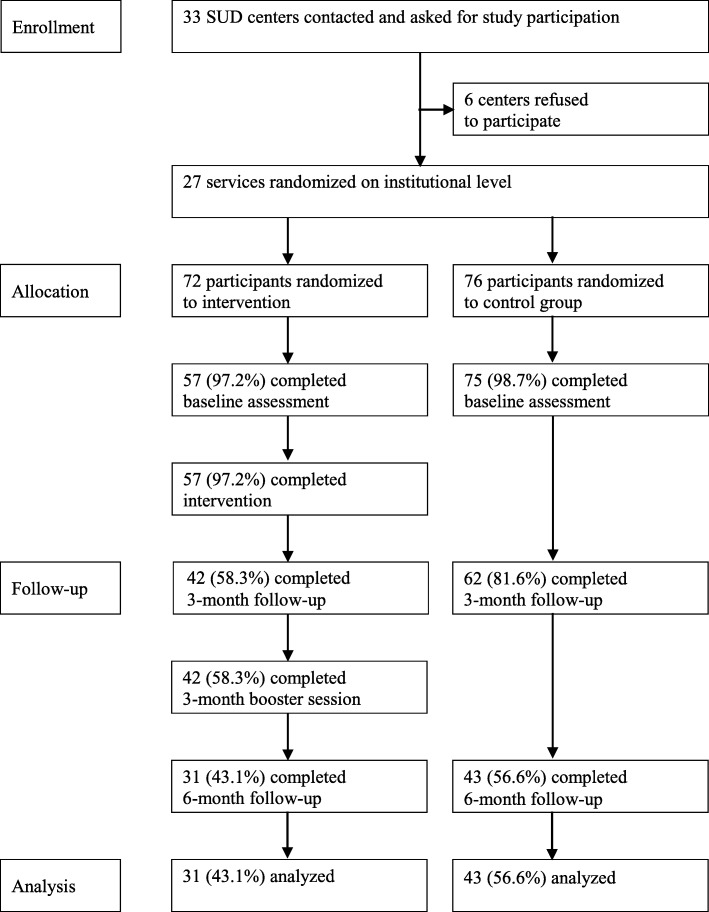
Flow of participants through the trial

Out of 33 SUD counseling centers contacted, the heads of 27 SUD centers, belonging to 11 different SUD organizations, were willing to participate in the study (Fig. 1). The heads of six counseling centers chose not to participate for different reasons, e.g., the counsellors had to leave work to participate in the training, another study was already being conducted at the center, or the topic of the study was perceived as not relevant for the counselors’ working practice. One hundred forty-eight SUD professionals of the 27 centers were cluster-randomized, by SUD organizations. Out of 148 randomized SUD health care professionals, 72 were allocated to the intervention group and 76 were allocated to the control group. One hundred thirty-two professionals could be assessed at baseline, including 57 (97.2%) professionals in the intervention group and 75 (98.7%) professionals in the control group. At 6-month follow-up, 74 professionals could be assessed, including 31 (54.4%) professionals in the training group, and 43 (56.6%) professionals in the control group.

### Intervention

The one-day ‘Learning How to Ask’ training includes eight 50-min units that cover the following topics: (1) Types and prevalence of traumatic events; (2) Effects of traumatic events on mental health; (3) Symptoms characterizing posttraumatic stress disorders; (4) Barriers to trauma inquiry; (5) Learning how to ask about traumatic events; (6) Learning how to respond to reports of traumatic events; (7) Documentation of traumatic events; and (8) Trauma-related resources available in the community. In the training, health care professionals are encouraged to reflect on their current routine practice of trauma inquiry and their barriers to inquiry. Participants learn basic knowledge about trauma and their consequences. Basic rules of asking about and responding to reports of traumatic events are discussed and practiced in role-plays.

### Procedure

Thirty-three German SUD centers of the federal states Hamburg and Schleswig-Holstein were contacted and informed about the study. Health care professionals were recruited from May 2014 to August 2014, and data were assessed between September 2014 and March 2015 before the intervention, at 3-month and at 6-month follow-up.

The health care professionals in the intervention group participated in the one-day ‘Learning How to Ask’ training on the inquiry of traumatic events and adequate response strategies. The training took place at the university medical center at which the study was conducted or at the SUD center if on-site training was preferred. The trainings were conducted by one experienced psychiatrist (last author) and/or one graduated psychologist (first author) in groups ranging from 5 to 16 professionals.

To standardize the content of the training, a power-point presentation covering the full training content was constructed and used for all trainings, and standardized instructions were used for all practical exercises. A short 1.5-h refresher training was conducted 3 months later. Professionals in the control group received no training during the data assessment period of the study, but received the same intervention after the data assessment had been completed. Professionals did not receive incentives for their study participation.

### Measures and primary outcomes

As no validated measure exists to assess health care professionals’ barriers to trauma inquiry, we constructed a questionnaire. Six common barriers to trauma inquiry were selected based on published research (see introduction) and discussions with SUD stakeholders: *‘Feeling uncomfortable when asking about traumatic events’, ‘Fear of offending the client when asking about traumatic events’, ‘Fear of retraumatizing the client when asking about traumatic events’, ‘Fear that client may terminate treatment when asking about traumatic events’, ‘Unsure whether authorities have to be informed when perpetrator is known’, ‘No trauma-specific treatment available in my local area’.* Professionals rated their level of agreement to these statements on a four-point Likert scale (0 = strongly disagree, 1 = somewhat disagree, 2 = somewhat agree, 3 = strongly agree). The primary outcomes were change over time from baseline in the six perceived barriers to trauma inquiry.

Inter-rater reliability was estimated for the six outcomes between the three measurement points (ICC, 2-way-mixed effects model) in the professionals that received no intervention, and was rated as good according to common standards [36]: ‘*Feeling uncomfortable when asking about traumatic events*’: ICC = .91; ‘*Fear of offending the client when asking about traumatic events*’: ICC = .81; ‘*Fear of retraumatizing the client when asking about traumatic events*’: ICC = .90; ‘*Fear that client may terminate treatment when asking about traumatic events*’: ICC = .80; ‘*Unsure whether authorities have to be informed when perpetrator is known*’: ICC = .75; ‘*No trauma-specific treatment available in my local area*’: ICC = .79).

Sociodemographic characteristics and potential confounders (professionals’ age, gender, migration background [i.e., immigration into Germany from another country, or born in Germany but immigration of at least one parent into Germany], professional group, duration working in the SUD center, previous trauma training within the last 3 years and type of substance use of clients [legal substances, illegal substances, or both] were also assessed by questionnaire.

### Sample size

Using a repeated-measures F test with a .05-level of significance and assuming a moderate effect size of 0.60 for self-report measures to evaluate the effect of a training [37], we estimated a sample size of *n* = 74 to achieve 80% power to detect a medium effect size of η^2^ = .059 [38]. Assuming an attrition rate of 40% at 6-month follow-up, we aimed to recruit 120 study participants.

### Randomization

The participants were randomized to the intervention or a control group at the level of the SUD organizations. This randomization level was chosen because professionals often worked in more than one counseling center of a provider; so randomizing at the level of the centers would have introduced contamination. The allocation schedule for the random assignment of the SUD organizations to the intervention or control group was generated by the randomization software DatInf RandList Version 1.2. Randomization was stratified by the number of health care professionals (< 20 vs. ≥ 20 employees). Allocation ratio was 1:1. No blocking was used within each of the strata. The randomization list was stored in a password-protected data file.

### Main analysis

All data provided by the participants of the study were included in the Intention-To-Treat analysis, regardless of whether they received the assigned treatment or not. The training condition was compared with the control condition on the amount of change from baseline at 3-month and 6-month follow-up. The primary outcomes were analyzed by linear mixed models (LMM), adjusted for the baseline values. All available cases for each variable were used in analysis. Missing values in the independent variables were not imputed, because there were no missing data in the independent variables used in this analysis.

The professionals and the SUD organizations were included in all final models as random effects to control for the repeated measurement or the randomization at the level of the SUD organizations, respectively. The SUD counseling centers were not included as an additional random effect in the final models, because this variable explained no additional variance of the outcomes. The fixed effects of the time point (3-month vs. 6-month) and the group (intervention vs. control) and their interaction term were included in the models to test whether the group had an effect on the outcome and whether this effect changed over time. If the group by time interaction term had no effect on the outcome, we followed the principle of parsimony and removed the interaction term from the model, as it can increase standard errors.

To control for potential confounding, variables that have been shown or are likely to be related to barriers to trauma inquiry (see introduction) were included in the LMMs: professionals’ age, gender, migration background (‘yes’ or ‘no’), professional group (‘social worker’, ‘psychologist’, ‘other profession’ or ‘trainee’), duration working in the SUD center (‘0 to <2 years’, ‘2 to <5 years’, ‘5 to <10 years’ or ‘more than 10 years’) and previous trauma training within the last 3 years (‘yes’ or ‘no’). Type of substance use of clients (‘predominantly legal substances’, ‘predominantly illegal substances’ or ‘legal and illegal substances balance each other’) was included in the analysis to control for potential confounding influences, because SUD stakeholder outlined this variable as associated with trauma inquiry (a focus group discussion on the content of the training was held prior to the start of the trial). All statistical analyses were conducted with STATA (Version 14.0, Stata Corp, College Station, Texas, USA).

### Sensitivity analysis

We conducted a sensitivity analysis to test whether the imputation of missing values in the outcomes would have changed the results. In this second analysis, missing data were imputed in the outcome variable (0.1% of the outcome data was missing at baseline, 1.9% at 3-month follow-up, and 0.5% at 6-month follow-up), using multiple imputation (MICE algorithm of STATA).

## Results

### Sample characteristics

The SUD professionals’ age ranged between 21 and 65 years (Table 1). The professionals of the training group were significantly younger than the professionals in the control group. About two third were trained as social education workers. About one third of the included SUD centers served clients with SUD related to both legal and illegal substances. They were in contact with 32 clients per month in both groups, on average. More than half of the professionals had been working for more than 5 years in the SUD center.

**Table 1:** Characteristics of Health Care Professionals

Characteristic	*n*	Intervention (*n* = 57)		*n*	Control (*n* = 74)		*p*
		*M*	*SD*		*M*	*SD*	
Age	57	42.9	12.2	74	47.0	9.2	.028
Number of clients/month	55	31.7	18.4	67	32.1	17.8	.900
		**f**	**%**		**f**	**%**	
Gender	57			74			.893
Female		34	59.6		45	60.8	
Male		23	40.4		29	39.2	
Migration background	57			74			.561
Yes		11	19.3		11	14.9	
No		46	80.7		61	82.4	
Unknown		0	0.0		2	2.7	
Professional group	57			74			.869
Social education worker		44	77.2		54	73.0	
Psychologist		6	10.5		10	13.5	
Other profession		4	7.0		6	8.1	
Trainee		3	5.3		4	5.4	
Duration working in center	56			74			.100
0 to <2 years		14	25.0		9	12.3	
2 to <5 years		12	21.4		10	13.5	
5 to <10 years		9	16.1		17	23.0	
≥ 10 years		21	37.5		38	51.4	
Previous trauma training	57			74			.321
Yes		20	35.1		20	27.0	
No		37	64.9		54	73.0	

Group comparisons were conducted with t-tests, Chi^2^-test or Fisher’s Exact test, depending on the measurement scale of the variable and the distribution of the cases

Out of the 132 professionals that completed baseline assessment, one professional of the control group did not report any data on barriers to trauma inquiry at baseline, at 3-month and at 6-month follow-up, and was therefore excluded from analysis. Means and standard deviations for the six measured barriers to trauma inquiry at baseline, at 3-month and at 6-month follow-up are reported in Table 2. At baseline, the professionals somewhat agreed with the statements indicating the six barriers to trauma inquiry, on average. Barriers to trauma inquiry were highest in both groups for ‘Feeling uncomfortable’, ‘Fear of offending the client’ and ‘Fear of retraumatizing the client’. At baseline, professionals in the intervention group reported significantly greater barriers to trauma inquiry regarding the ‘Fear that client may terminate treatment’ and ‘Unsure whether authorities have to be informed when perpetrator is known’.

**Table 2:** Means and Standard Deviations of Perceived Barriers to Trauma Inquiry at Baseline, 3-Month and 6-Month Follow-up

Barriers to trauma inquiry	Baseline						*p*	3-month follow-up						6-month follow-up					
	Intervention (*n* = 57)			Control (*n* = 74)				Intervention (*n* = 42)			Control (*n* = 61)			Intervention (*n* = 31)			Control (*n* = 43)		
	*n*	*M*	*SD*	*n*	*M*	*SD*		*n*	*M*	*SD*	*n*	*M*	*SD*	*n*	*M*	*SD*	*n*	*M*	*SD*
‘Feeling uncomfortable’	57	1.40	0.68	74	1.30	0.77	.154	41	1.20	0.63	60	1.30	0.74	31	1.10	0.67	43	1.30	0.75
‘Fear of offending the client’	57	1.50	0.71	74	1.30	0.86	.121	42	1.10	0.71	60	1.20	0.88	31	0.97	0.71	43	1.30	0.85
‘Fear of retraumatizing the client’	57	1.30	0.71	74	1.30	0.79	.949	42	0.67	0.61	60	1.20	0.81	31	0.81	0.48	42	1.40	0.76
‘Fear that client may terminate treatment’	57	1.10	0.62	74	0.83	0.60	.025	42	0.69	0.64	60	0.83	0.64	31	0.68	0.60	42	0.93	0.64
‘Unsure whether authorities have to be informed when perpetrator is known’	57	1.20	0.92	74	0.81	0.91	.034	42	0.88	0.86	61	0.74	0.85	31	0.45	0.72	43	0.88	0.85
‘No trauma-specific treatment available in my local area’	57	1.40	0.86	74	1.10	0.87	.093	42	1.00	0.91	60	1.20	0.84	31	1.00	0.82	43	1.20	0.93

0 = I strongly disagree, 1 = I somewhat disagree, 2 = I somewhat agree, 3 = I strongly agree

### Drop-out analysis

No significant differences in the six barriers to trauma inquiry were found at baseline between the professionals with complete assessments and those without complete assessments in the intervention or control group, respectively: ‘Feeling uncomfortable when asking about traumatic events’: completer *M* = 1.30, *SD* = 0.75 vs. non-completer *M* = 1.36, *SD* = .73; *t*(129) = − 0.37, *p* = .712; ‘Fear of offending the client’: completer *M* = 1.33, *SD* = 0.76 vs. non-completer *M* = 1.46, *SD* = .81; *t*(128) = − 0.76, *p* = .447; ‘Fear of retraumatizing the client’: completer *M* = 1.40, *SD* = 0.72 vs. non-completer *M* = 1.29, *SD* = .75; *t*(129) = 0.73, *p* = .468; ‘Fear that client may terminate treatment’: completer *M* = .80, *SD* = 0.61 vs. non-completer *M* = .98, *SD* = .62; *t* (129) = − 1.41, *p* = .161; ‘Unsure whether authorities have to be informed when perpetrator is known’: completer *M* = .77, *SD* = 0.90 vs. non-completer *M* = 1.03, *SD* = .93; *t*(129) = − 1.37, *p* = .174; and ‘No trauma-specific treatment available in my local area’: completer *M* = 1.10, *SD* = 0.89 vs. non-completer *M* = 1.25, SD = 0.87; *t*(129) = − 0.82, *p* = .416.

### Main analysis

**Fig. 2:**
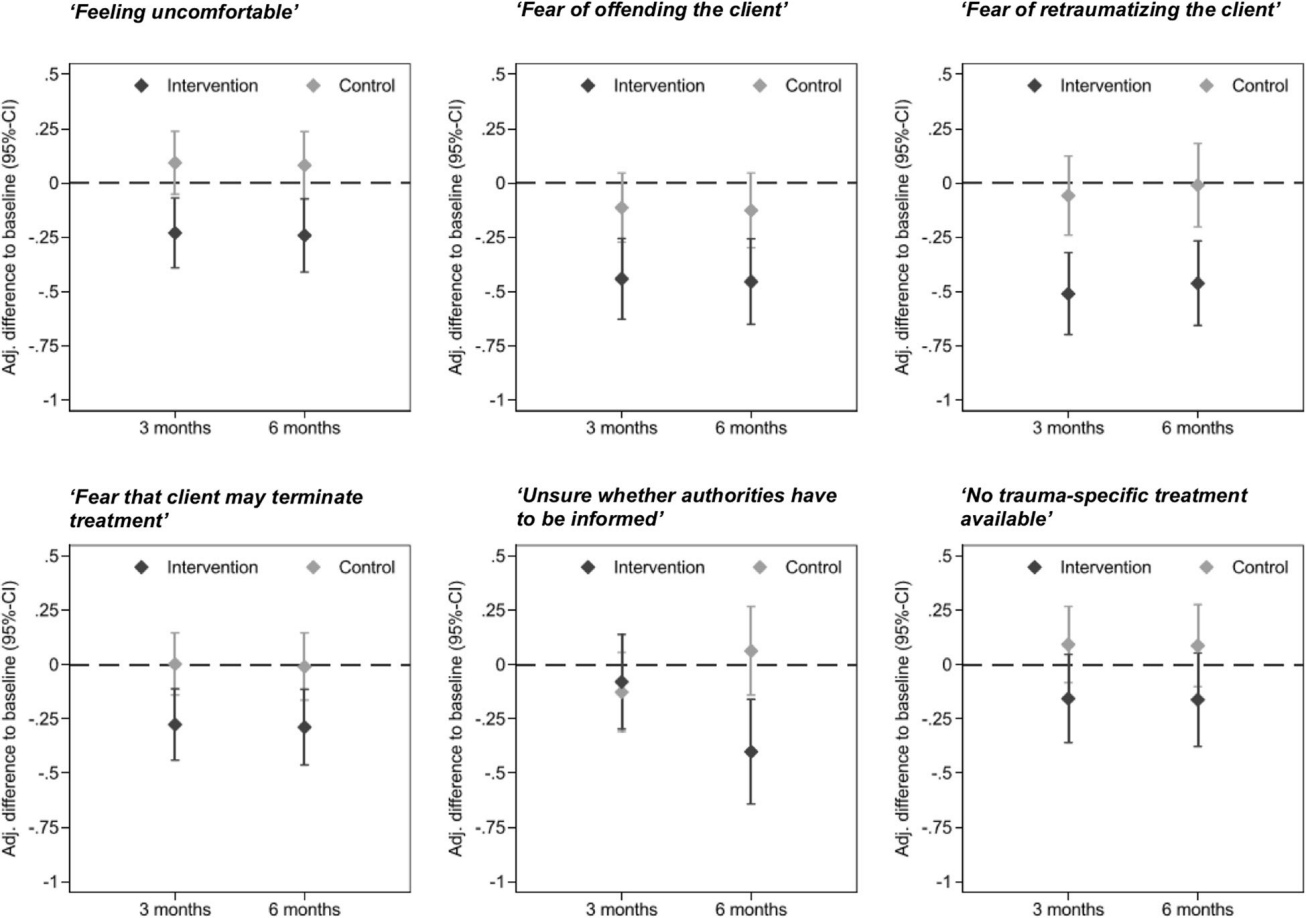
Covariate Adjusted Change from Baseline in Barriers to Trauma Inquiry at 3-Month and 6-Month Follow-up

The covariate-adjusted change from baseline in the six barriers to trauma inquiry in the intervention and control group are shown in Fig. 2.

***‘Feeling uncomfortable when asking about traumatic events’.***

The intervention group showed a significantly greater decrease in *‘Feeling uncomfortable’* than the control group (Fig. 2; *b* = − 0.32, 95% CI [− 0.52, − 0.12], *p* = .002). There was no significant effect of the time point (3-month follow-up vs. 6-month follow-up) on the outcome, indicating that the intervention effect remained stable over time. None of the potential confounders had a significant effect on ‘*Feeling uncomfortable’*.

***‘Fear of offending the client when asking about traumatic events’.***

The intervention group showed a significantly greater decrease in *‘Fear of offending the client*’ than the control group (*b* = − 0.33, 95% CI [− 0.56, − 0.09], *p* = .006). The time point (3-month follow-up vs. 6-month follow-up) did not significantly affect the outcome, indicating that the intervention effect remained stable.

Professionals who had participated in a previous trauma training showed a significantly greater reduction in their ‘*Fear of offending the client’*, compared with those who had not participated (*b* = 0.26, 95% CI [0.02, 0.51], *p* = .037). None of the potential confounders had a significant effect on the outcome.

***‘Fear of retraumatizing the client when asking about traumatic events’.***

Compared with the control group, the intervention group showed a significantly greater decrease in the *‘fear of retraumatizing the client’* (*b* = − 0.45, 95% CI [− 0.69, − 0.22], *p* < .001). There was no significant effect of the time point on the outcome. None of the potential confounders had a significant impact on the outcome.

***‘Fear that client may terminate treatment when asking about traumatic events’.***

Compared with the control group, the intervention group showed a significantly greater decrease in the ‘*Fear that client may terminate treatment’* when asking about traumatic events (*b* = − 0.28, 95% CI [− 0.49, 0.07], *p* = .009). There was no significant effect of the time point on the outcome, indicating that the intervention effect remained stable over time. None of the potential confounders significantly affected the outcome.

***‘Unsure whether authorities have to be informed when perpetrator is known’.***

A group-by-time interaction effect was found for the change in feeling *‘Unsure whether authorities have to be informed when perpetrator is known’* when asking about traumatic events (z = − 3.14, CI [− 0.83, − 0.19], *p* = .002). The intervention group showed a greater decrease (*b* = − 0.32, 95% CI [− 0.56, − 0.08], *p* = .009) than the control group (*b* = − 0.19, 95% CI [− 0.02, 0.40], *p* = .075). Compared to the control group, the intervention group did not show a greater decrease in *‘Unsure whether authorities have to be informed‘*at 3-month follow (*b* = 0.05, 95% CI [− 0.25, 0.34], *p* = .753), but showed a greater decrease at 6-month follow-up (*b* = − 0.46, 95% CI [− 0.79, − 0.14], *p* = .005).

Gender significantly predicted the change in their feeling *‘Unsure whether authorities have to be informed’* when asking about traumatic events (*b* = − 0.30, 95% CI [− 0.578, 0.04], *p* = .024), with males in both groups showing a greater change than females.

***‘No trauma-specific treatment available in my local area’.***

The training group showed a slightly greater decrease in the belief that *‘No trauma-specific treatment’* is *‘available’* than the control group, but the difference was not significant (*b* = − 0.25, 95% CI [− 0.51, − 0.01], *p* = .059). There was no effect of the time point of assessment on the outcome, indicating that the intervention effect remained stable over time.

Age significantly predicted the change in the belief that *‘No trauma-specific treatment’* is *‘available’* (*b* = − 0.25, 95% CI [− 0.40, − 0.10], *p* = .001)*,* with younger age being related to a greater reduction in this belief.

### Sensitivity analysis

A sensitivity analysis was conducted to test whether the imputation of missing values in the outcomes would have changed the results. The sensitivity analysis showed that all effects found in the main analysis remained significant, and all non-significant results remained non-significant (see Additional file 1).

## Discussion

In this study, we examined for the first time whether a training in trauma inquiry, combined with a short refresher session, is able to reduce perceived barriers to trauma inquiry that are often reported by health care professionals, such as the fear to harm the client [15, 16, 39]. This study is, therefore, a novel and important contribution to the research field of trauma-informed care in SUD settings.

We found that five of six barriers to trauma inquiry more greatly decreased among the trained professionals than among the untrained professionals from baseline to 6-month follow-up. According to our results, a one-day training in trauma inquiry might be effective in reducing these barriers to trauma inquiry that are frequently reported by professionals. There is no earlier study that assessed the effects of an intervention in reducing barriers to trauma inquiry with which we could directly compare our results. In a pilot study that evaluated the original training program in trauma inquiry in a small sample [23], it was found that the training significantly increased confidence in asking about abuse, which might be related to the reduction of barriers to trauma inquiry.

In this study, the ‘*Fear of retraumatizing the client when asking about traumatic events’* was directly addressed in the training by discussing this fear, explaining the difference between short screening questions to assess the clients’ trauma history and trauma-focused psychotherapy, and by presenting research results demonstrating that questions about traumatic events do not harm the client [40, 41]. These interventions seem to have reduced the professionals’ fear of retraumatizing the client.

The training could also significantly reduce the ‘*Fear of offending the client when asking about traumatic events*’. Some of the trained professionals reported that their own experience of being asked about traumatic events during the role-plays of the training was helpful to reduce this fear.

The perceived barrier *‘No trauma-specific treatment available in my local area’* was addressed in the training by providing information about possible local treatment options for traumatized clients, e.g., counseling centers specializing in supporting victims of abuse, or outpatient and inpatient treatment services for clients affected by trauma. This information may have helped to reduce the professionals’ belief that no trauma-specific treatment is available. However, it should be noted that not only perceived but also structural barriers to trauma-specific treatment exists. For example, the average waiting time for an initial meeting for a psychotherapy is more than 3 months in Germany [42], and trauma-specific treatment services are not available for all patients that need it [43, 44]. Such structural barriers cannot be removed by trainings, but need changes in the health care system. In addition, the implementation of trauma-informed and trauma-focused approaches in SUD facilities may decrease barriers related to access to services by targeting the trauma and also thereby improving SUD treatment outcomes and reducing risk of relapse.

The fear that the ‘*Client may terminate treatment when asking about traumatic events’* also reduced more among the trained professionals than the untrained professionals. With increasing practice in asking about traumatic events, the trained professionals might have experienced that the clients continued their treatment despite of being asked.

Feeling *‘Unsure whether authorities have to be informed when perpetrator is known’* was significantly reduced among the trained professionals at 6-month follow-up, but not at 3-month follow-up. This result might be explained by the professionals’ increasing familiarity with trauma cases, resulting in greater knowledge in legal obligations related to traumatic events. As the training did not lead to a reduction in the unsureness whether authorities have to be informed when perpetrator is known 3 months after the training, information about legal obligations should be addressed more in detail in further trainings of trauma inquiry.

Although about one third of the professionals reported that they had participated in some form of training related to the topic of trauma within the last three years, most of the professionals reported barriers to the inquiry of traumatic events. Trainings that provide information about trauma and its consequences might be ineffective in changing perceived barriers to trauma inquiry and trauma inquiry behavior. Tailored trainings that target both perceived barriers to trauma inquiry and trauma inquiry behavior seem necessary to change relevant attitudes and behaviors. The results of this study showed that the ‘Learning How to Ask Training’ is able to reduce barriers to trauma inquiry. Earlier published results on this trial [33] indicated that the intervention was also effective in increasing professionals’ trauma inquiry behavior in the short term. The change of perceived barriers to trauma inquiry, in addition to behavioral change, seems critical for the long-term implementation of the newly learned behavior, as professionals may fall back into their old behavior if perceived barriers (e.g., the belief that asking about traumatic events is harmful to the client) could not be reduced by the training.

### Strengths and limitations

A strength of our study is that we evaluated the effectiveness of a one-day training in reducing barriers to trauma inquiry using a cluster-randomized controlled trial design, including two follow-up assessments. We reached a sample of SUD outpatient health care professionals that varied in age and professional experience. Besides these strengths, our study results are limited by our reliance on self-report measures that might be related to socially desirable responding. Another limitation of this study is the use of self-constructed measures to assess trauma inquiry behavior. This was done because no validated measure of barriers to trauma inquiry exists. Future studies should develop and validate questionnaires of trauma inquiry behavior that cover different aspects of barriers to trauma inquiry behavior that can then be used in future studies.

Randomizing the SUD health care professionals at the level of the SUD organizations is another limitation of our study. This was done to minimize bias related to treatment contamination caused by professionals that work in more than one SUD center. While this approach minimized bias related to treatment contamination, this randomization approach might have produced bias related to cluster-randomization. The SUD organizations might differ in their working cultures regarding trauma inquiry, their working settings, and the proportion of clients exposed to traumatic events. These variables might be related to the professionals’ perceived barriers to trauma inquiry. However, our data analysis revealed that the effect of the SUD organizations explained no variance in the outcomes.

A limitation of our study is the different baseline completion rates in the intervention and control group (79% intervention group vs. 99% control group). The professionals that did not complete the baseline assessment were those who did not attend the training. The different attrition rates in the two groups might be related to differences in the outcomes that are not caused by the intervention. Significant attrition occurred from baseline to 6-month follow-up. The professionals that dropped out of the study might have changed the results of the study, as they might systematically differ from the professionals that could be reassessed. However, we found that completers did not differ from non-completers in barriers to trauma inquiry at baseline.

It should also be noted that we conducted six separate linear mixed models for analysis. Multiple testing without adjusting the *p*-value increases the risk of false findings of significant effects. Another methodological issue that can be discussed is our approach to remove a non-significant time by group interaction term from the linear mixed model, following the principle of parsimony. This method might have changed the results of the analysis. However, we compared the results of our final models with the respective models including the non-significant interaction term, and found that the results did not change in terms of a significant effect.

Finally, professionals were not blinded to group assignment, which may have produced biased results.

### Suggestions for future research

In order to better understand the mechanisms through which barriers can be reduced in SUD health care professionals, future studies might examine which elements of the training are most effective in reducing barriers to trauma inquiry. Future studies might also address provider-level barriers to trauma-inquiry rather than individual barriers. It should also be noted that we evaluated a training in trauma inquiry in health care professionals working in SUD settings. However, it is likely that the training is also effective for health care professionals working in other health care settings, which might be examined in further studies.

## Conclusions

Systematic inquiry of traumatic events in SUD services is essential to increase detection of traumatic events in survivors in order to adequately address trauma-related treatment needs. This cluster-randomized study found that a one-day training in trauma inquiry, combined with a short refresher training, reduced SUD health care professionals’ perceived barriers to trauma inquiry 3 and 6 months after the initial training. According to these results, SUD professionals’ frequently reported barriers to trauma inquiry can be reduced with short trainings, which may enhance the detection of traumatic events in survivors.

## Additional file

Additional file 1: Sensitivity Analysis. (DOCX 81 kb)

## Abbreviations

PTSD: Posttraumatic stress disorder
SUD: Substance use disorder
