# Supplementary material for: Reducing barriers to trauma inquiry in substance use disorder treatment – a cluster-randomized controlled trial
Source: Subst Abuse Treat Prev Policy. 2019 May 29;14:23. doi: 10.1186/s13011-019-0211-8 (PMC6541998; doi:10.1186/s13011-019-0211-8)
Supplement: Supplementary file 1 — Sensitivity Analysis. (DOCX 81 kb) [file 13011_2019_211_MOESM1_ESM.docx]

**Additional file 1: Sensitivity Analysis**

**‘Feeling uncomfortable when asking about traumatic events’**

|  | With Multiple Imputation |  | Without Multiple Imputation |  |
| --- | --- | --- | --- | --- |
|  | b / 95%-CI | p-value | b / 95%-CI | p-value |
| **Treatment** (ref: No) |  |  |  |  |
| - Intervention | -0.31 | 0.003 | -0.32 | 0.002 |
|  | -0.51,-0.11 |  | -0.52,-0.12 |  |
| **Follow-Up** (ref: 3 months) |  |  |  |  |
| - 6 months | 0.00 | 0.997 | -0.01 | 0.870 |
|  | -0.18,0.18 |  | -0.15,0.13 |  |

**‘Fear that client may terminate treatment’**

|  | With Multiple Imputation |  | Without Multiple Imputation |  |
| --- | --- | --- | --- | --- |
|  | b / 95%-CI | p-value | b / 95%-CI | p-value |
| **Treatment** (ref: No) |  |  |  |  |
| - Intervention | -0.34 | 0.001 | -0.28 | 0.009 |
|  | -0.55,-0.14 |  | -0.49,-0.07 |  |
| **Follow-Up** (ref: 3 months) |  |  |  |  |
| - 6 months | 0.02 | 0.813 | -0.01 | 0.867 |
|  | -0.16,0.20 |  | -0.15,0.13 |  |

**‘Fear of retraumatizing the client when asking about traumatic events’**

|  | With Multiple Imputation |  | Without Multiple Imputation |  |
| --- | --- | --- | --- | --- |
|  | b / 95%-CI | p-value | b / 95%-CI | p-value |
| **Treatment** (ref: No) |  |  |  |  |
| - Intervention | -0.35 | 0.007 | -0.45 | 0.001 |
|  | -0.60,-0.10 |  | -0.69,-0.22 |  |
| **Follow-Up** (ref: 3 months) |  |  |  |  |
| - 6 months | 0.10 | 0.314 | 0.05 | 0.494 |
|  | -0.10,0.30 |  | -0.09,0.19 |  |

**‘Unsure whether authorities have to be informed when perpetrator is known’**

|  | With Multiple Imputation |  | Without Multiple Imputation |  |
| --- | --- | --- | --- | --- |
|  | b / 95%-CI | p-value | b / 95%-CI | p-value |
| **Interaction term** | -0.50 | 0.022 | -0.51 | 0.002 |
|  | -0.93,-0.07 |  | -0.83,-0.19 |  |
| **Treatment** (ref: No) |  |  |  |  |
| - Intervention | 0.00 | 0.984 | 0.05 | 0.753 |
|  | -0.37,0.37 |  | -0.25,0.34 |  |
| **Follow-Up** (ref: 3 months) |  |  |  |  |
| - 6 months | 0.19 | 0.204 | 0.19 | 0.075 |
|  | -0.10,0.49 |  | -0.02,0.40 |  |

**‘Fear of offending the client when asking about traumatic events’**

|  | With Multiple Imputation |  | Without Multiple Imputation |  |
| --- | --- | --- | --- | --- |
|  | b / 95%-CI | p-value | b / 95%-CI | p-value |
| **Treatment** (ref: No) |  |  |  |  |
| - Intervention | -0.34 | 0.006 | -0.33 | 0.006 |
|  | -0.58,-0.10 |  | -0.56,-0.09 |  |
| **Follow-Up** (ref: 3 months) |  |  |  |  |
| - 6 months | -0.02 | 0.868 | -0.01 | 0.877 |
|  | -0.22,0.18 |  | -0.17,0.15 |  |

**‘No trauma-specific treatment available’**

|  | With Multiple Imputation |  | Without Multiple Imputation |  |
| --- | --- | --- | --- | --- |
|  | b / 95%-CI | p-value | b / 95%-CI | p-value |
| **Treatment** (ref: No) |  |  |  |  |
| - Intervention | -0.25 | 0.077 | -0.25 | 0.059 |
|  | -0.53,0.03 |  | -0.51,0.01 |  |
| **Follow-Up** (ref: 3 months) |  |  |  |  |
| - 6 months | -0.01 | 0.932 | -0.01 | 0.951 |
|  | -0.27,0.25 |  | -0.18,0.17 |  |
